# Improvement and Evaluation of Loop-Mediated Isothermal Amplification for Rapid Detection of *Toxoplasma gondii* Infection in Human Blood Samples

**DOI:** 10.1371/journal.pone.0169125

**Published:** 2017-01-05

**Authors:** Xi-meng Sun, Yong-sheng Ji, Xian-yong Liu, Mei Xiang, Guang He, Li Xie, Jing-xia Suo, Xun Suo

**Affiliations:** 1 National Animal Protozoa Laboratory & College of Veterinary Medicine, China Agricultural University, Beijing, China; 2 State Key Laboratory for Agrobiotechnology, China Agricultural University, Beijing, China; 3 Key Laboratory of Zoonosis of Ministry of Agriculture, China Agricultural University, Beijing, China; 4 The Second Hospital of Jilin University, Changchun, China; Universidade Federal de Pelotas, BRAZIL

## Abstract

Loop-mediated isothermal amplification (LAMP), an attractive DNA amplification method, was developed as a valuable tool for the rapid detection of *Toxoplasma gondii*. In this study, species-specific LAMP primers were designed by targeting the AF146527 sequence, which was a conserved sequence of 200- to 300-fold repetitive 529 bp fragment of *T*.*gondii*. LAMP reaction system was optimized so that it could detect the minimal DNA sample such as a single tachyzoite or 10 copies of recombinant plasmid. No cross-reactivity was found when using DNA from other parasites as templates. Subsequently, a total of 200 human blood samples were directly investigated by two diagnostic methods, LAMP and conventional PCR. Fourteen of 200 (7%) samples were positive for *Toxoplasma* by LAMP (the primers developed in this study), whereas only 5 of 200 (2.5%) were proved positive by conventional PCR. The procedure of the LAMP assay was very simple, as the reaction would be carried out in a single tube under isothermal conditions at 64°C and the result would be read out with 1 h (as early as 35 min with loop primers). Thus, this method has the advantages of rapid amplification, simple operation, and easy detection and would be useful for rapid and reliable clinical diagnosis of acute toxoplasmosis, especially in developing countries.

## Introduction

Toxoplasmosis is a common parasitic disease caused by infection of *Toxoplasma gondii* (*T*. *gondii*), which infects a variety of mammals including rodents, cats, and humans [1]. Infection of the parasite can be acquired by eating raw meat containing tissue cysts, or food and water contaminated by oocysts [2]. Acute infection can result in pregnant women transplacental transmission to the fetus and severe damage to the fetus, which eventually causes neonatal malformations, neurological damage, blindness, or stillbirth [3]. *T*. *gondii*is typical of short duration and self-limiting when signs and symptomsare present.The hostpersists in cyst form without related clinical symptoms. However, persistent or recrudescent clinical manifestations, life-threatening consequences may result in newborns and immunocompromised hosts, such as encephalitis, myocarditis, and chorioretintis [4].

Diagnosis of toxoplasmosis is important for the control of the disease. Laboratory diagnosis has historically relied on one of the below several ways in acute *T*. *gondii* infection. For example, the parasitological and histological method is to isolate the tachyzoite stage from blood or body fluids such as tissue culture. The immunologic methods are the detection of *T*. *gondii* antigens or specific antibodies, such as indirect flurorescent-antibody test and enzyme-linked immunosorbent assays [5,6]. The molecular biological method as PCR is to amplify the specific *T*. *gondii* genes or DNA fragments [7]. The modified agglutination test, immunochromatographic test and the real-time PCR have also been developed as diagnostic methods for *T*. *gondii* infection [8–10]. Despite these advances, diagnosis of *T*. *gondii* infection remains unsatisfactory because serological methods are time-consuming, and false-positive results may occur due to extra-parasitic materials during parasite antigen preparation [11], and sometime cannot differentiate early infections [12]. Although PCR-based techniques have been developed recently, they are limited due to long reaction time and the expensive cost of the apparatus [7,13]. Therefore, it is still necessary that we develop the cost-effective and rapid detection methods for *T*. *gondii*.

Loop-mediated isothermal amplification (LAMP) originally developed by Notomi is a newly introduced technique that amplifies target nucleicacids with high sensitivity, specificity, efficiency, and rapidity under isothermal conditions [14]. The LAMP method uses a DNA polymerase called *Bst* polymerase with displacement activity and a set of 4 especially designed primers that recognize a total of 6 conserved and distinct sequences of the target DNA. So far, LAMP has been applied for the diagnosis of various parasitic diseases, including trypanosomosis [15–17], piroplasmosis [18]. In addition, LAMP has been used for the detection of *Cryptosporidium parvum* oocysts [19]. LAMP assay has also been used for the detection of *Toxoplasma gondii* based on the B1 and TgOWP genes in water samples [20], diagnosis of toxoplasmosis based on the SAG1, SAG2 and B1 genes from human blood samples [21]. Recently, AF146527 fragment (GenBank AccessionNo. AF146527), which is a 200- to 300- fold repetitive 529 bp fragment of *T*. *gondii*, has been utilized for the development of a very sensitive and specific PCR for diagnosis [8, 22], and even in LAMP assay on swine DNA samples [23].

In the present study, we improved the LAMP method for *T*. *gondii* detection based on AF146527 sequence. We obtained the optimal reaction condition as the temperature of 64°C, reaction time for 1 hour, the Mg^2+^ and betine concentration of 3.0–6.0mM and 1.0 mM, respectively. Moreover, the LAMP method was proved to be very specific and sensitive in detecting *T*. *gondii* in DNA samples and human blood samples.

## Materials and Methods

### Parasite culture

*T*. *gondii* RH strain was used in our study. Tachyzoites were maintained in Vero cell monolayerand cultured in Dulbecco Modified Eagle Medium (HyClone, USA) supplemented with 5% fetal calf serum(FCS, HyClone, USA) at 37°C, in 5% CO_2_ atmosphere for 3 to 4 days. Tachyzoites were purified with a 27-gage needle syringe and a 5.0 μm pore filter (Millipore,MA, USA) by previously described method [24]. Purified tachyzoites were stored at -70°C for further use.

### Clinical samples

A total of 200 blood samples were collected from pregnant volunteers in the Second Hospital ofJilin University, China. The blood sampling was conducted by experienced staff in this hospital to prevent unnecessary risk of exposure to blood and to avoid adverse events for participates.The following conditions were used as inclusion criteria for participation: The age had a limit of 22 to 40 years old; they were in contact with or had been raising dogs, cats and other pets; they were not taken any toxoplasma related medical examination. Oral informed consent from these anonymous participates was recorded by us in the present of a witness. The study was conducted after being approvedby the Ethics Review Board of China Agricultural University, and the consent procedure in this study was also approved by IRB.

Blood samples were collected in tubes containing EDTA and citrate and sent to our laboratory where they were stored at 4°C until processing. All samples were handled under aseptic conditions.

### LAMP reaction system

Four primers were used for the LAMP assay targeting six conserved region of the AF146527 sequence (GenBank AccessionNo. AF146527) of *T*. *gondii*. The outer forward primer (F3), outer backward primer (B3), forward inner primer (FIP) and backward inner primer (BIP) were designed using the Primer Explorer program (http://primerexplorer.jp/elamp4.0.0/index.html). Two sets of the four primers are listed in Table 1. The LAMP reactionmixture (25 μL) contained 2μLof the extracted DNA, 1.6μM of primers FIP and BIP, 0.2 μM of primers B3 and F3, 1 μLof BstDNA polymerase (New England Biolabs), 1.0 M betaine (Sigma-Aldrich), 1.6mM deoxynucleoside triphosphate (dNTP), and 2.5 μL of 10×buffer (New England Biolabs) [20 mM Tris-HCl (pH 8.8), 10 mM KCl, 2.0mM MgSO_4_, 10 mM (NH4)_2_SO_4_, 0.1% Tween-20]. The LAMP reaction was performed for 30 to 60 min at 65°C and was stopped by heat inactivation at 80°C for 2 min. Electrophoresis with 1.5% agarose gel was used to analyze 20 μL of the LAMP products, followed by ethidium bromide staining and photography.

**Table 1:** Nucleotide sequences of 2 groups of LAMP primers (AF and RE) designed in this study.

*Primer*	*Sequence (5’-3’)*
**AF-F3 **	**GTTGGGAAGCGACGAGAG**
**AF-B3**	**ACAGTGCATCTGGATTCCTC**
**AF-BIP**	**CCGGTGTCTCTTTTTCCACCCTTCGGAGAGGGAGAAGATGTT**
**AF-FIP**	**CCTCGTGGTGATGGCGGAGATCCCTTCGTCCAAGCCTC**
**RE-F3**	**GTTGGGAAGCGACGAGAG**
**RE-R3**	**CAGTGCATCTGGATTCCT**
**RE-BIP**	**CTCGTGGTGATGGCGGAGATTTTCGTCCAAGCCTCCGACTCTG**
**RE-FIP**	**CGGTGTCTCTTTTTCCACCCTTTTTTCGGAGAGGGAGAAGATGTTT**

### Analytical sensitivity and specificity of LAMP assay

The primer sequences 529F (5’-CGCTGCAGGGAGGAAGACGAAAGTTG-3’) and 529R (5’-CGCTGCAGACACAGTGCATCTGGATT-3’) were selected to specifically amplify the AF146527 DNA sequence in *T*. *gondii* genome as described previously [22]. The DNA fragment was inserted into pEASY-T1 Simple Cloning Plasmid (Transgene Co., China). The plasmid was evaluated by Nucleic Acid/Protein Analyzer DU^®^ 800 (Beckman Coulter, USA) and the copies of AF146527 sequence were calculated using the following formula:

Copies/mL = Conc. of Plasmid (g/mL)×Avogadro's Constant/Molecular Weight of Plasmid (g/mol)

Molecular Weight of Plasmid (g/mol) = Average Molecular Weight of Bases×Total Number of Bases in Plasmid

The plasmids were diluted 10-fold serially as 10^9^ copies to 1 copy to determine the sensitivity of LAMP assay. Besides, tachyzoites of *T*. *gondii* RH strain harvested from the cell culture were counted on a hemocytometer and diluted as 40, 20, 15, 10, 5 and 1 tachyzoite(s) in 200 μL of fresh human blood. For LAMP assay, genomic DNA was extracted from the 200 μL sample solution with a DNA Extraction Kit (Tiangen, Beijing, China). The reactions were performed at least twice. In addition, 1 tachyzoite in PBS was used as a template of LAMP assay (without DNA extraction). At the same time, DNA derived from *Eimeria tenella*, *Cryptosporidium parvum*, *Neospora caninum* and *Trypanosoma evansi* was used to check the specificity of *T*. *gondii* LAMP assay.

### Conventional PCR

In brief, PCR was carried out in a 25 μL reaction volume which contained 2μL DNA template, 0.5 μL forward primer and 0.5 μL reward primer (529F and 529R as described before), 12.5μL 2× TaqMix (Transgene, Beijing), 9.5 μL double distilled water. The amplification was performed with a PCR machine (T-Gradient, Biometra®), that consisted of initial denaturation at 94°C for 5 min, followed by 30 cycles of 94°C for 45 sec, primer annealing at 55°C for 30 sec, extension at 72°C for 1 min and a final extension at 72°C for 10 min. PCR products were electrophoresedin a 1.2% agarose gel and stained with ethidium bromidefor visualization.

### Clinical sensitivity of LAMP assay

The clinical sensitivity and specificity of the LAMP assay were determined using 200 blood samples. Genomic DNA was extracted from 100 μL of blood samples with a DNA Extraction Kit (Tiangen, Beijing,China).The purified DNA was dissolved in 30 μL of double-distilled water for subsequent LAMP assay and conventional PCR.

## Results

### Optimization of LAMP assay

**Fig 1:**
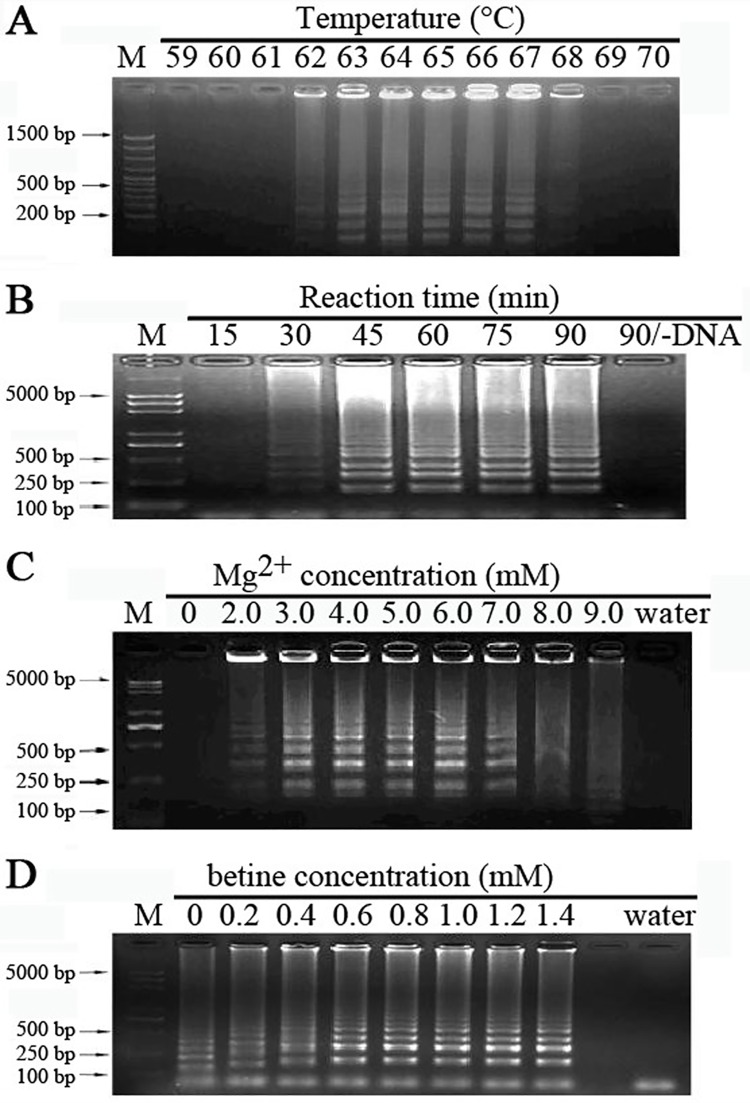
Optimization ofLAMP reactions. LAMP reaction were carried out using genomic DNA from tachyzoites under various conditions, as different reaction temperatures (A), reaction times (B), Mg^2+^ concentrations (C) and betine concentrations (D). Lane (M), DNA ladder.

The LAMP method is a rapid and convenient method for target DNA fragment detection. Like other DNA amplification methods, the reaction is also related to several factors, including temperature, time etc. As shown in Fig 1A, LAMP reaction was set up at different temperatures from 59 to 70°C. It is shown that the strongest signal was obtained when the reaction system was incubated at 63 to 64°C, whereas almost no signal at 59–62°C or over 67°C. According to the proper temperature of *Bst* DNA polymerase of 64°C, the optimal temperature of LAMP reaction was selected as 64°C. Then the LAMP reaction was lasted for different times from 15 min to 90 min. The LAMP reaction needed at least 45 min, and the product reached maximum when lasted for 60 min (Fig 1B). So the proper reaction time would be 1 hour. Mg^2+^ could enhance the efficiency of the *Bst* DNA polymerase. Mg^2+^ should keep at a proper level in the LAMP reaction system, since that the precipitation generated during the reaction would affect read out of the result with lower Mg^2+^, and too higher Mg^2+^ level would decrease the specificity of LAMP reaction. Obvious DNA band was seen when the concentration of Mg^2+^ was 3.0–6.0mM (Fig 1C). Finally, the optimal concentration of betine was checked. With the betine concentration over 0.6 mM, the target fragment got well amplified, which is consistent with the previous report, which betine could improve the stability of long length DNA and the specificity of LAMP reaction [14]. Due to the inhibitory effect of large amount betine, the most suitable concentration of betine is 1.0 mM (Fig 1D).

### Specificity of LAMP reaction

**Fig 2:**
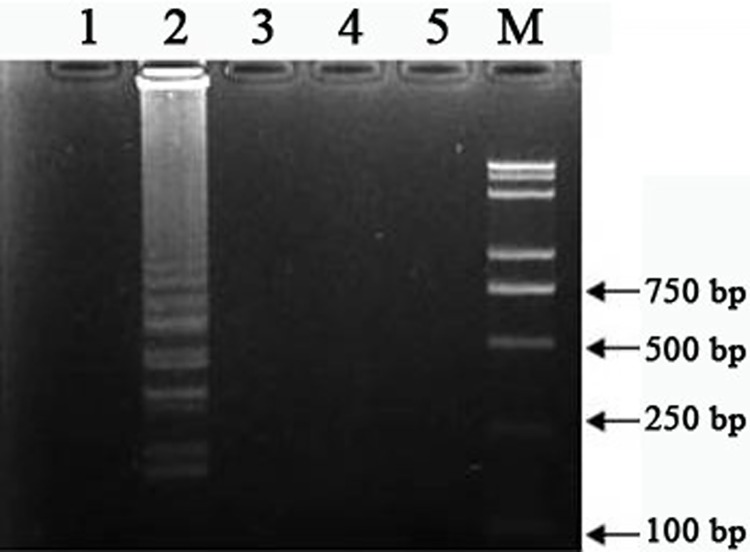
Specificity of LAMP. LAMP reaction was monitored for DNA amplification of *Eimeriatenella (lane 1)*, *Toxoplasma gondii (lane 2)*, *Trypanosoma evansi (lane 3)*,*Cryptosporidium parvum (lane 4)*and *Neosporacaninum (lane 5)*by gel electrophoresis. Lane (M), DNA ladder.

The designed four LAMP primers, F3/B3/FIP/BIP (Table 1), were used to amplify the AF146527 fragment from various DNA samples from *T*. *gondii*, and some other protozoan parasites including *E*. *tenella*, *C*. *Parvum*, *N*. *caninum* and *T*. *evansi*. First, in reaction containing *T*. *gondii* DNA, the target fragment was clearly seen (Fig 2, lane 2). While using DNA from other protozoa as the template, no such target fragment was obtained (Fig 2, lane 1 and lanes 3–5). It is suggested that LAMP assay with target specific primers showed high specificity in detection of *T*. *gondii*.

### Sensitivity of LAMP assay

**Fig 3:**
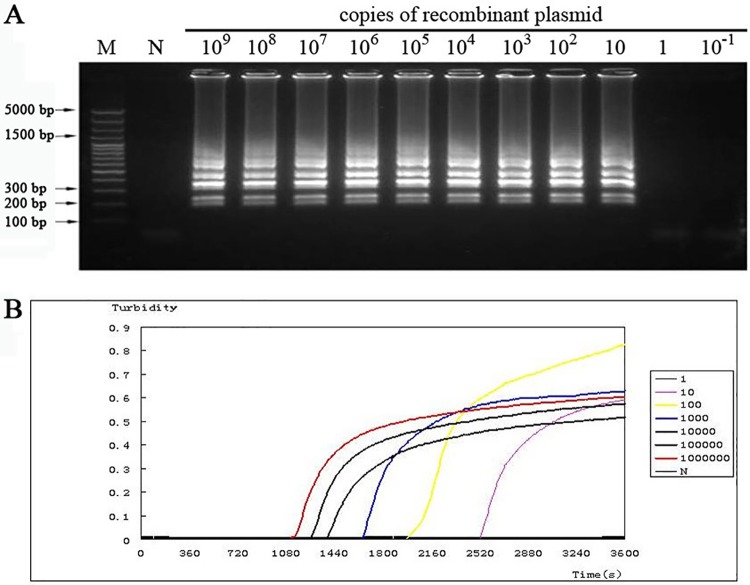
The sensitivity of LAMP was checked. (A) LAMP reaction with different copies of the recombinant plasmid as the template.Lane (M), DNA ladder. (B) LAMP reaction with different copies of the recombinant plasmid was monitoredon the LoopampRealtimeTuribidimeter.

The sensitivity of the LAMP assay was analyzed by detecting the DNA amplification in conditions containing different copies of DNA template. The recombinant plasmid containing AF146527 fragment was diluted serially, and then the obtained dilution such as 10^9^ copies to 1 copy of plasmidswasused as the template for the LAMP assay. As shown in Fig 3A, only 10 copies of the recombinant plasmid as the template, the target DNA fragment would be amplified in high efficiency. When the reaction system contained only 1 copy or even less of the recombinant plasmid, there was no DNA product for target fragment.

Moreover, the turbidity change was also monitored. The turbidity began to increase early at about 30 min when there contained 10^6^ copies of the recombinant plasmid (Fig 3B, red line). With the decrease of plasmid copies, the increasing of turbidity occurred later. The turbidity increased at late as 42 min in the condition of only 10 copies of the recombinant plasmid as the template (Fig 3B, purple line). There was no increase in turbidity even after 1h when only 1 copy of the recombinant plasmid was in the reaction system(Fig 3B). There was no obvious difference in maximum turbidity at the end of the reactions with different plasmid copies from 10^6^ to 10^1^.

**Fig 4:**
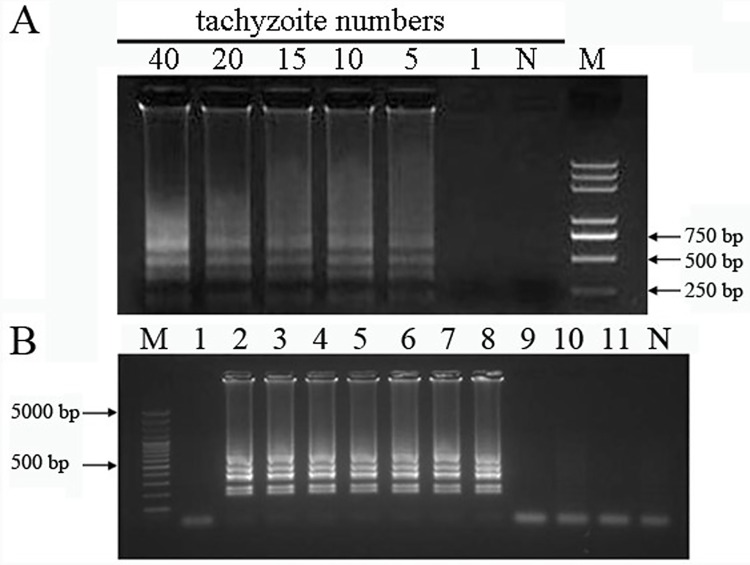
Sensitivity of the LAMP method. LAMP reactions were carried out using genomic DNA from various tachyzoite(s)mixed with 200 μL fresh human blood sample (A) and genomic DNA from single tachyzoite in PBS (B) as the template.

The sensitivity of LAMP was also checked in conditions that the template was the genomic DNA extracted from different numbers of tachyzoites. As shown in Fig 4A, the amplified DNA fragments were seen when using genomic DNA from at least 5 tachyzoites. In the condition with genomic DNA from only 1 tachyzoite, although there was no DNA product (Fig 4A), the amplified target fragment was seen in seven out of eleven reactions (Fig 4B, lanes 2–8).

From above, it is suggested that the LAMP assay was very sensitive in detection of *Toxoplasma* even with only 10 copies of the target fragment or only 5 tachyzoites in the sample.

### Comparison between LAMP and conventional PCR

A total of 200 clinical samples were subjected to the conventional PCR and LAMP assays. After analyzing via the PCR assay, 5 (2.5%) and 195 (97.5%) of the samples were judged to be positive and negative with *T*. *gondii*, respectively (Table 2). Also, the PCR products of the 5 positive samples were sequenced and showed 100% identity with the target sequence (Data not shown). Then both the previous positive and negative samples were analyzed by LAMP assay with different primers, including Xuan-LAMP [23], AF-LAMP, RE-LAMP (this article, Table 1), etc. As shown in Table 2, the previous 5 positive samples were all proved to be positive with *Toxoplasma* via AF-LAMP, RE-LAMP and Xuan-LAMP assays, but fewer positives were detected via LAMP with other LAMP primers (Table 2). Among the 195 negative samples analyzed by the conventional PCR, another 8 and 9 samples were proved to be positive with *Toxoplasma* checked by AF-LAMP and RE-LAMP, which resulted in the total detection ratio of 6.5% and 7%, respectively. However, using the Xuan-LAMP assay, there was a surprising number, 170 out of 195 were proved to be positive with *Toxoplasma* (Table 2), which suggested that the Xuan-LAMP might be too hypersensitive for toxoplasma detection. For other LAMP assays [20–21], only one to three samples out of 5 PCR-positive samples were detected to be positive by B1-LAMP assay and B1/SAG1/SAG2-LAMP assays, although 3 out of 195 negatives were proved to be positive via SAG2-LAMP. So the detection rate using other LAMP primers was lower than the conventional PCR (Table 2).

**Table 2:** Comparison of the conventional PCR and LAMP for detection of *T*.*gondii* in blood samples from pregnancy.

		*No*. *(%) with Xuan primers*^*a*^		*No*. *(%) with AF primers*		*No*. *(%) with RE primers*		*No*. *(%) with B1 primers*^*b*^		*No*. *(%) with Toxo B1 primers*^*c*^		*No*. *(%) with SAG1 primers*^*b*^		*No*. *(%) with SAG2 primers*^*b*^	
		+	−	+	−	+	−	+	−	+	−	+	−	+	−
**No. (%) with PCR**	**+**	**5 (2.5)**	**0**	**5 (2.5)**	**0**	**5 (2.5)**	**0**	**3 (1.5)**	**2 (1)**	**1 (0.5)**	**4 (2)**	**3 (1.5)**	**2 (1)**	**3 (1.5)**	**2 (1)**
	-	**170 (85)**	**25 (12.5)**	**8 (4)**	**187 (93.5)**	**9 (4.5)**	**186 (93)**	**0**	**195 (97.5)**	**0**	**195 (97.5)**	**0**	**195 (97.5)**	**3 (1.5)**	**192 (96)**
**Total percent (%)**		**87.5**	**12.5**	**6.5**	**93.5**	**7**	**93**	**1.5**	**98.5**	**0.5**	**99.5**	**1.5**	**98.5**	**3**	**97**

^**a**^Xuan primers: Zhanget al. Experimental Parasitology. 2009,122:47–50.

^**b**^B1,SAG1,SAG2 primers: Yeeet al.Journal of Clinical Microbiology, 2010,48:3698–3702.

^**c**^Toxo B1 primers: Isaiaet al. Diagnostic Microbiology and Infectious Disease, 2008,62: 357–365.

## Discussion

In this study, we improved the LAMP method for *T*.*gondii* detection, a highly specific and sensitive diagnostic system. The reaction conditions were screened for the most optimal as the temperature being 64°C, reaction time 1 hour, the Mg^2+^ and betine concentrations 3.0–6.0 mM and 1.0 mM, respectively. The LAMP method was proved to be very specific for detection of *T*. *gondii*. Moreover, the LAMP method was much more sensitive in detecting *T*. *gondii* than other methods such as the conventional PCR.

Since the invention of the LAMP assay, it has been successfully established in diagnosing diseases caused by viruses [25–27], bacteria [28] and parasites [15–16, 19]. Moreover, LAMP is used for detection of toxoplasma by amplification of various *T*. *gondii* genes, as TOXO B1, TgOWP, SAG1, SAG2 and AF146527 fragment. However, almost the same general reaction conditions were used for these genes or fragments except a little variation with the reaction temperature as 65°C or 63°C [20–21, 23]. In order to get the highest efficiency of LAMP assay based on the AF146527 fragment, reaction conditions were tested in this study. Using the optimal condition, the target fragment has been well amplified (Fig 1).

Mover, we determined the specificity of the LAMP assay through screening heterologous genomic DNA from other parasites, no cross-reactivity with *E*. *tenella*, *C*. *parvum*, *N*. *caninum* and *T*. *evansi* DNA samples was detected. The detection limit of this LAMP assay was 5 tachyzoites, which was less sensitive than the nested PCR target at B1 gene [21], when using pure *T*. *gondii* DNA as a template. However, when plasmids containing single copy of AF146527 sequence were used, the detection limit was 10 copies of the target sequence. The difference between these two limits may be due to DNA losses during *T*.*gondii* genomic DNA extraction.

For the 200 human blood samples from pregnant women, the LAMP assay and conventional PCR were both tested. All the five PCR-positive blood samples were proved by DNA sequencing, which suggested that there was no false-positive result. The above 5 samples were also be detected as positive by our LAMP assay (AF-LAMP and RE-LAMP), as well as Xuan-LAMP assay. Only one to three samples out of 5 PCR-positive samples were detected to be positive by B1-LAMP assay and B1/SAG1/SAG2-LAMP assays, suggesting their lower sensitivity. To amplify B1/SAG1/SAG2 genes, the LAMP assay was carried out according to their reported general reaction conditions [20–21]. So, the lower sensitivity of B1/SAG1/SAG2-LAMP assay might be caused by the LAMP reaction efficiency, as well as the character of different sample types [20–21]. For the 195 PCR-negative samples, several samples were judged as positive via our LAMP assay indicating higher sensitivity than the conventional PCR. Xuan-LAMP showed higher sensitivity (78/91) than the conventional PCR (70/91) in the detection of *T*. *gondii* [23]. Here the 170 samples in 195 PCR-negative samples were determined as positive by Xuan-LAMP assay, which indicated a surprisingly high sensitivity. This might due to the tissue specificity.

## Conclusions

Our results have demonstrated that the AF146527-based LAMP assay could be applied for detection of *T*. *gondii* with the detection rate of at least 10 copies of the target DNA fragment from 5 tachyzoites. Also our LAMP method showed higher sensitivity and valid rate in the processing of clinical samples. Although detection rate varies among different LAMP assays, the LAMP method still has its advantages in specificity, rapidity, sensitivity and easiness for wide application both in veterinary and medical fields. More works need to be done to further improve the LAMP method in diagnostic approach for Toxoplasmosis, and even for other pathogens.
